# Severe teratozoospermia and its influence on pronuclear morphology, embryonic cleavage and compaction

**DOI:** 10.1186/1477-7827-9-37

**Published:** 2011-03-22

**Authors:** Dara S Berger, Faten AbdelHafez, Helena Russell, James Goldfarb, Nina Desai

**Affiliations:** 1 Cleveland Clinic Foundation, Department of OB-GYN, 26900 Cedar Road, Cleveland, Ohio 44122, USA; 2 Eastern Virginia Medical School, School of Health Professions, Department of OB-GYN, 601 Colley Avenue, Norfolk, Virginia 23507, USA

## Abstract

**Background:**

Fertilization, cell division and embryo development depend on genomic contributions from male and female gametes. We hypothesize that teratozoospermic sperm influences early embryo development and embryo compaction.

**Methods:**

We conducted a retrospective analysis of embryos derived from intracytoplasmic sperm injection (ICSI) cycles. Two hundred thirty-five consecutive ICSI cycles were included in the study; all treatment was provided at the Cleveland Clinic Fertility Center. Patient cycles were divided by sperm morphology based on Kruger's strict criteria: Group A, embryos where teratozoospermic sperm (0-2% normal) were used for ICSI and Group B, embryos where dysmorphic sperm (5-13% normal) were used for ICSI. All cycles analyzed were of patients doing day 3 embryo transfers. Outcome measures assessed included pronuclear (PN) pattern, syngamy, early cleavage, cell number, rate of compaction and blastulation of embryos left in culture and not transferred on day 3.

**Results:**

A total of 1762 embryos were analyzed. PN patterns were similar in Group A and Group B embryos. No differences were noted in syngamy, cleavage, cell number or blastulation rate. Studying the development of embryos in culture after day 3 transfer revealed a difference in the timeline for compaction. By day 4, 25% of Group A embryos had compacted compared to 36% in Group B (P = 0.0007). There was no difference found between Group A and Group B embryos in regards to blastulation.

**Conclusions:**

We did not find an association between sperm morphology and clinical outcomes. The impact of teratozoospermia may be masked in ICSI cycles where fertilization, implantation rate and clinical pregnancy rate are the primary outcome measures. However, by examining the timeline of development, we were better able to discern a potential paternal effect at critical transition points from fertilization through activation.

## Background

Intracytoplasmic sperm injection (ICSI) has allowed for the use of extremely teratozoospermic sperm specimens for *in vitro *fertilization (IVF). Fertilization by direct injection of the sperm into the oocyte circumvents the usual process for combining male and female genomes. While oocytes supply the cytoplasmic environment necessary for sperm microtubule function, the sperm is responsible for the centrosome, which gives rise to microtubules [1]. Human reproduction involves several critical steps including fertilization of the oocyte, syngamy of male and female pronuclei, cleavage, genomic activation, blastulation and finally implantation. If these developmental processes are hindered, the embryo will arrest and a viable pregnancy will not be achieved.

Paternal effect on early embryonic development and subsequent fertility is an area of great interest. Abnormal sperm morphology can be an indicator of one or more other defects including damaged DNA, chromosomal abnormalities and centriole deficiency [2,3]. Data suggests that morphologically abnormal sperm and/or DNA fragmented sperm have a negative impact on fertilization and embryo quality, even when ICSI is performed [4-7]. Evidence of a strong paternal effect on early embryonic development and blastocyst formation *in vitro *has been reported [8]. The relationship between sperm morphology and IVF/ICSI cycle outcomes still however remains controversial. In a more recent study, French et al. observed that severe teratozoospermia had no effect on blastulation [9].

The earliest morphological assessment of embryos in the laboratory occurs at the time of fertilization check. The presence of two pronuclei (PN) signals the combining of male and female genome in the developing zygote [10]. Pronuclear morphology and arrangement of nucleolar precursor bodies (NPB) provide the earliest information on sperm:egg interaction. The NPB number, size and distribution have been correlated to embryo quality, development and IVF outcome [10-15]. Specific PN morphology patterns have also been associated with an increase in implantation and pregnancy rates [11,12,14-18]. Another critical point in development is embryonic genomic activation, when genes from maternal genome are no longer expressed [19]. During *in vitro *culture this is marked by signs of embryonic compaction usually observed on day 3 or day 4 of culture [20,21].

In this investigation we compare embryonic development from zygote to blastocyst in patients with severe teratozoospermia to those with less severely dysmorphic sperm. We postulate that this may be a good model to study paternal contribution to early embryonic development because with severe teratozoospermia, chromosomal anomalies and inherent DNA damage may interfere with early steps of zygote formation, syngamy, cleavage, genomic activation and blastulation. These data will also allow us to better understand the contribution of sperm morphology assessment to outcomes with ICSI.

## Methods

### Patients

We retrospectively analyzed embryo morphology along with IVF cycle outcomes. Embryos were generated by ICSI, using sperm from patients with varying levels of dysmorphic morphology, including teratozoospermia. This study was conducted under an approved IRB and involved analysis of data collected from patients undergoing treatment at the Cleveland Clinic Fertility Center between June 2006 and June 2008. All patients included were < 37 years of age to minimize advanced maternal age factors. Cycles were further divided into subgroups based on sperm morphology assessment using Kruger's strict criteria. Two subpopulations were created, Group A consisted of 110 patients including 901 embryos from severely teratozoospermic sperm (0-2% normal morphology), and Group B consisted of 125 patients including 861 embryos from less dysmorphic sperm (5-13% normal forms). Patients with sperm morphology not falling into Group A or Group B were excluded. Cycles with insufficient sperm (< 1 million/ml) were excluded.

### Sperm morphology assessment and preparation for ICSI

Sperm specimens were evaluated for total sperm count, motility, progression and sperm morphology during IVF semen analysis. Sperm were stained using Diff-Quik (Dade Behring, Newark, Delaware) and graded on the basis of Kruger's strict criteria [22]. Percent normal forms were determined for each specimen after scoring 100 sperm. Uniformity of grading in the laboratory was routinely monitored for inter technician variation. Density gradient centrifugation over a 90% layer of Isolate (Irvine Scientific, Santa Ana, California) was used to prepare sperm for ICSI.

### IVF protocol

Patients were down regulated using a long luteal leuprolide acetate (Lupron; TAP, Pharmaceuticals, Lake Forest, Illinois) protocol, followed by gonadotrophins for ovulation induction. Ultrasound guidance was used to transvaginally aspirate and collect oocytes. Under an oil overlay, ooctyes were cultured in microdrops of human tubal fluid (HTF) (LifeGlobal, Guilford, Connecticut) supplemented with human serum albumin (10 mg/mL) (Cooper Surgical Inc., Trumball, Connecticut). Culture dishes were incubated at 37°C with 5.5% CO_2_. ICSI was performed on mature oocytes 3-4 hours after retrieval. Sperm selection for ICSI was based on identification of the most normal looking sperm in the available sperm specimen that also exhibited some motility. Any observed variations between Group A and Group B embryos were therefore most likely reflective of intrinsic differences in the initial sperm specimen since the methodology for sperm selection did not differ between Group A and Group B. Fertilization was checked 18-20 hours after oocyte injection. Zygotes were classified into seven morphologic patterns based on number and distribution of nucleoli within the pronucleus, consistent with Kruger's strict criteria. Fertilized oocytes were cultured individually until day 3 in microdrops of HTF supplemented with 10% Synthetic Serum Substitute (SSS) (Irvine Scientific, Santa Ana, California).

Embryo development was monitored daily. All patients included had a day 3 transfer. Day 3 embryo grading was based primarily on cell number, percent fragmentation and signs of increased cell:cell adherence or compaction [23]. Pronuclear pattern, syngamy and early cleavage were also given consideration in evaluating the embryo. Non-transferred embryos of good quality (6-8 cells, < 15% fragmentation) were cryopreserved. All other embryos remained in culture for an additional 2-3 days; blastocysts of good quality were subsequently frozen on either day 5 or 6. Embryo transfer was performed under ultrasound guidance using a Wallace catheter. Pregnancy testing was done 15-16 days after the embryo transfer. Clinical pregnancy was confirmed by the presence of a fetal heart on ultrasound at 6-8 weeks of pregnancy.

### Data collection and analysis

Patient embryos were grouped on the basis of sperm morphology used for the ICSI procedure, Group A versus Group B. The primary outcome measures included fertilization rate after ICSI, number of embryos transferred, rate of blastocyst formation, implantation rate and clinical pregnancy. The disposition of embryos derived from sperm samples with severe teratozoospermia (Group A, 0-2% normal) was compared to those embryos created from sperm with less severe dysmorphisms (Group B, 5-13% normal).

Two hundred fifty-six transfers were performed on day 3. In order to compare how poor sperm quality influences embryo development, we grouped patients according to degree of sperm dysmorphisms and analyzed PN patters, syngamy achievement, presence of early cleavage, cell number at day 2 and day 3, rate of compaction, rate of blastulation and embryo destiny (i.e. whether the embryo was transferred, frozen or discarded).

Fertilization rates were calculated by dividing the number of oocytes that fertilized normally by the number of oocytes subjected to the ICSI procedure. PN morphology assessments were recorded per embryo at the time of fertilization check, 18-20 hours after ICSI was performed. Blastulation rate was calculated by dividing the number of embryos that blastulated by the number of embryos in culture past day 3. The implantation rate was calculated by dividing the total number of fetal hearts by the total number of embryos transferred. Clinical pregnancy was determined by the presence of a fetal heart on ultrasound at 6 weeks.

Means and standard deviations were used to summarize continuous indices such as patient age, oocytes fertilized, number of embryos transferred and sperm concentration. Pronuclear pattern, syngamy and early cleavage and development were expressed as a percentage of embryos examined. Chi-square test and the student T-test were applied as appropriate to compare summary data as well as development rates of embryos. PN patterns were further evaluated using Cochran-Mantel-Haenszel (CMH) statistics to determine significance between the seven patterns observed and the outcome measures of interest. P values less than 0.05 were considered to be statistically significant.

## Results

A total of 1762 embryos derived from 258 oocyte retrievals of patients who fit the criteria for inclusion in our study. The 235 women in this study were between 24 and 36 years of age. Embryonic development was compared between embryos derived from sperm samples with severe teratozoospermia, Group A defined as embryos where ICSI was performed with sperm 0-2% normal forms, and less dysmorphic samples, Group B with embryos where ICSI was performed with sperm 5-13% normal forms. This data set allowed us to examine the effect of severe teratozoospermia versus dysmorphic sperm on embryo development including cleavage, compaction and the ability of embryos to blastulate.

Fertilization rate was not affected by the morphology of the initial sperm specimen. Moreover implantation and clinical pregnancy rates were like-wise unaffected by sperm morphological characteristics. The clinical pregnancy rate for day 3 transfer patients in Group A was 55% versus 53% in Group B (Table 1).

**Table 1:** Patient data

	**Group A**	**Group B**
Number of patients	110	125
Number of transfers	121	135
Average age	31.88 ± 2.739	32.59 ± 2.64
Average oocytes per retrieval	12.99 ± 5.78	13.23 ± 6.84
Average mature oocytes per retrieval	9.93 ± 4.98	9.21 ± 4.37
Number of embryos	901	861
Fertilization rate	79%	78%
Average number of cells in transferred embryos, observed on day 3	7.50 ± 1.35	7.34 ± 1.50
Average embryos transferred	2.14 ± 0.39	2.12 ± 0.44
Implantation rate	33%	33%
Clinical pregnancy rate	55%	53%

Group A: ICSI was done using severe teratozoospermic sperm, 0-2% normal morphology, Group B: ICSI was done using dysmorphic sperm, 5-13% normal morphology. P values for data above all > 0.05.

The influence of severe teratozoospermia at the earliest stage of zygote development, i.e. formation of pronuclei, was investigated. The pronuclear patterns observed in embryos transferred, frozen or discarded were compared between Group A and Group B (Table 2). Regardless of sperm type, the majority of zygotes exhibited 3-7 large nucleoli that were polarized and aligned (Pattern OP). The second most prevalent arrangement observed was Pattern 2. Interestingly, Patterns 4 and 5, usually associated with poorer embryo potential [10] were not represented at a higher frequency in embryos arising from teratozoospermic sperm specimens, P > 0.05.

**Table 2:** PN pattern in embryos

	**Group A**				**Group B**			
		
**PN pattern**	**Embryos transferred n = 248**	**Embryos frozen on day 3 n = 162**	**Embryos in extended culture n = 471**	**Blastulated embryos from extended culture**	**Embryos transferred n = 254**	**Embryos frozen on day 3 n = 151**	**Embryos in extended culture n = 432**	**Blastulated embryos from extended culture**
		
OP	50%	54%	46%	18%	45%	43%	51%	20%
O	14%	15%	10%	3%	11%	12%	10%	4%
1	2%	1%	2%	1%	2%	2%	2%	1%
2	19%	16%	22%	6%	20%	24%	22%	8%
3	0%	1%	3%	1%	4%	2%	1%	0%
4	8%	9%	12%	4%	9%	11%	9%	1%
5	6%	4%	5%	1%	9%	7%	5%	2%

Group A: ICSI was done using severe teratozoospermic sperm, 0-2% normal morphology, Group B: ICSI was done using dysmorphic sperm, 5-13% normal morphology. P values for data above all > 0.05.

Table 3 compares benchmarks of *in vitro *development of embryos in Group A and Group B. Syngamy and early cleavage proceed similarly in both groups of embryos. When comparing cell number, 64% of Group A embryos were 6 cells or greater as compared to 66% in Group B. Embryonic blastulation was also not affected by morphology of sperm in the original specimen. With day 3 transfer cycles, 'spare' embryos were cultured for an additional 2-3 days. We did not see a significant negative impact from sperm morphology on blastocyst formation. Embryo utilization, i.e. percentage of zygotes transferred and frozen in the two groups was compared. The percentages of embryos either frozen or transferred were not significantly different between Group A and Group B. Severe teratozoospermia was not associated with a difference in overall embryo utilization.

**Table 3:** Embryo development and utilization data

	**Group A**	**Group B**	**P value**
Total embryos	901	861	
**Embryonic development, D1-D3**			
Syngamy (at 25 hours)	39%	34%	0.0516
Early cleavage (at 25 hours)	9%	9%	1.0000
Cleavage (D2 at 48 hours)	98%	99%	0.3833
Embryos with ≥ 6 cells (D3)	64%	66%	0.4543
**Compaction**			
Embryos compacting (D4)	25%	36%	0.0007
**Blastulation**			
Total embryos in extended culture	471	432	
Overall blastulation rate	34%	38%	0.3297
**Overall utilization**			
Embryos transferred	28%	30%	0.3865
Embryos frozen	34%	36%	0.3401

Group A: ICSI was done using severe teratozoospermic sperm, 0-2% normal morphology, Group B: ICSI was done using dysmorphic sperm, 5-13% normal morphology. Yates-corrected Chi^2 ^P values given above.

Most notable was observations of embryonic compaction between Group A and Group B (Table 3). The percentage of embryos exhibiting compaction was analyzed on day 4 of culture. We found that significantly fewer embryos from Group A had undergone compaction compared to Group B embryos (P = 0.0007). Interestingly, although slower to compact, by the end of the culture interval (day 5/6) Group A blastulation was 34% compared to Group B 38% blastulation, P > 0.05; blastulation rates were not significantly different even though compaction rates showed a delay in Group A.

## Discussion

The focus of the study was to characterize early development in embryos derived from severely teratozoospermic specimens and compare the pattern of development to embryos derived from less dysmorphic sperm. Such observations might help us better understand paternal contribution to embryo development *in vitro*. Our data suggested that embryonic compaction, an early indication of genome activation, was the only parameter significantly influenced by sperm morphology. It appeared that embryos derived from teratozoospermic sperm were slower to compact, but by day 5/6 of culture, blastulation rates were similar in the two groups. We also noted severe teratozoospermia did not impair implantation or clinical pregnancy rates, which is in concordance with findings from Hotaling et al's 2010 meta-analysis of teratozoospermia and clinical pregnancy after IVF [24].

The impact of overall sperm morphology assessment on IVF outcomes, especially in ICSI cases, has been the focus of many idiopathic infertility studies but remains controversial. Kruger et al. proposed that morphological classification of sperm based on strict criteria can be used to predict fertilization outcomes [25]. Kruger's strict criteria states sperm specimens with less than 14% normal forms have decreased ability to fertilize. Others have proposed further grouping on the basis of sperm morphology into good prognosis 4-14% normal forms and poor prognosis < 4% normal forms [7,26,27]. Our study concentrated on embryos derived from two populations of suboptimal sperm. We specifically chose to examine embryos derived from extremely teratozoospermic sperm specimens with 0-2% normal forms and 5-13% normal forms. This was done in order to accentuate differences between the groups. Using 5-13% normal forms as cut-off for dysmorphic sperm and eliminating the middle group with 3-4% normal forms, we excluded specimen bordering Group A and Group B. While these sperm groupings may be unconventional, our aim was to study the impact of poor sperm morphology on embryo development and not necessarily to compare good sperm morphology to poor sperm morphology.

A direct correlation between abnormal sperm morphology and sperm function has been difficult to establish. A meta-analysis studying outcomes with conventional IVF strongly supports the diagnostic value of critical morphology assessment in sperm specimens [28]. Specifically the type of sperm morphological defect may be correlated to whether there is a connection between morphology and chromosome abnormalities. Furthermore, FISH studies suggest a greater frequency of aneuploidy with certain forms of teratozoospermia such as globospermia, enlarged head syndrome and polymorphic teratozoospermia [29]. Other reports contradict these finding and claim abnormal sperm morphology is a poor predictor of cycle outcomes [30-32].

Sperm DNA fragmentation and a correlation with sperm morphology is also relevant when investigating teratozoospermia and embryonic development. While the embryo may be able to repair low levels of DNA damage introduced by the sperm nucleus [33,34], if the sperm DNA is damaged beyond repair, complete and accurate transmission of genetic information to the embryo may become compromised. In addition, amorphous sperm heads have been associated with an elevated degree of DNA fragmentation [35]. Using intracytoplasmic morphologically selected sperm injection (IMSI) to choose sperm has shown abnormal nuclei usually have a lower fertility potential than sperm with normal nuclei [36].

With ICSI, the normal barriers to fertilization are transcended allowing even the most teratozoospermic specimens to successfully create a zygote. DNA fragmentation in morphologically normal looking sperm has also been shown to negatively impact on ICSI cycle outcomes and embryo quality [6]. Thus sperm morphology may not by itself be an accurate predictor of the competency of the DNA within the sperm itself, nor chromosomal status. Paternal effect on early embryogenesis is often overlooked, yet the sperm centrosome is directly involved in forming the sperm aster and organizing the mitotic spindle. While human oocytes do not contain centrioles, the sperm contains two. Microtubules, which extend from centrioles, are responsible for proper pronuclei movement and fusion [37], as well as pronuclei alignment [38]. Centriolar defects, or an absence of centrioles, are most often associated with compromised sperm [39,40] and may affect microtubule formation [41]. We therefore had expected to see differences in PN patterns between Group A and Group B.

The earliest stage post-fertilization to assess the impact of sperm on zygote development is pronuclear formation. Pronuclear morphology and nucleolar organization have been correlated to embryo quality [10,42]. Our expectation was that if teratozoospermia was associated with an increase in DNA defects within the sperm, or centriolar defects, pronuclear patterns would be negatively affected. Zygotes with abnormal PN patterns such as those with less than 3 NPB in at least one PN (Pattern 4) or those in which there are both polarized and non-polarized NPB (Pattern 5), have been suggested to have lower pregnancy potential [13]. These zygotes with abnormal PN patterns have further been reported to more frequently result in chromosomally abnormal embryos than those with normal PN patterns [43]. With ICSI cases, the use of teratozoospermic sperm specimens did not appear to increase the percentage of abnormal PN patterns. The percentage of zygotes with polarized NPB (Pattern OP) believed to be predictive of good embryo quality was similar between the two sperm test groups, Group A and Group B.

Human embryo genomic activation occurs on or around day 3. Expression of zygote transcripts has been shown to begin at approximately the 4 to 8 cell stage [19,44]. An early morphologic indicator of embryonic genome activation is compaction, the increase of cell:cell adherence, also occurring around the 8-cell stage [45]. Examining the day 4 data collected showed a delay in the timing of compaction in the cohort of embryos derived from Group A sperm, Table 3. A greater percentage of embryos compacted by day 4 in Group B, although the final blastulation rates between sperm groups remained similar. One potential explanation for the significant decrease in compaction rates we observed is that there is delayed genome activation in the Group A embryos possibly attributable to the severe teratozoospermia in the specimens used for ICSI. Compaction occurs as a result of an intricate series of events dependent upon many factors including but not limited to E-cadherin and catenein complexes, junction components, protein kinase C and the PAR complex [20,45-47]. With so many variables involved, our observation correlating compaction rates with genomic activation would greatly benefit from further studies.

In conclusion, we did not find an association between sperm morphology and clinical outcomes. The impact of teratozoospermia may be masked in ICSI cycles where fertilization, implantation rate and clinical pregnancy are the primary outcome parameters measured. Through sequential analysis of embryos from fertilization through blastulation we hoped to achieve a better understanding of paternal influence on the different stages of embryonic development. Contrary to our expectations, we did not observe an early paternal effect at the point of pronuclear formation nor an effect on blastulation rate. We did however find a measurable difference in compaction, which we feel strongly warrants further investigation into the paternal effect on embryonic activation. A detailed step-wise analysis of morphology and the timeline of development in embryos arising from severely teratozoospermic sperm versus sperm with less dysmorphisms has not been previously reported. Design of a prospective study with day 5 transfers may be extremely valuable in further studying paternal effect on zygotic activation.

## Competing interests

The authors declare that they have no competing interests.

## Authors' contributions

DB designed the project, collected and analyzed the data and drafted the manuscript. FAH helped format the manuscript. HR participated in the study design. JG aided with the manuscript edits and overall content. ND conceived of the study, supervised the design and coordination of the manuscript. All authors read and approved the final manuscript.
